# Batrachotoxin acts as a stent to hold open homotetrameric prokaryotic voltage-gated sodium channels

**DOI:** 10.1085/jgp.201812278

**Published:** 2019-02-04

**Authors:** Rocio K. Finol-Urdaneta, Jeffrey R. McArthur, Marcel P. Goldschen-Ohm, Rachelle Gaudet, Denis B. Tikhonov, Boris S. Zhorov, Robert J. French

**Affiliations:** 1 Department of Physiology & Pharmacology and Hotchkiss Brain Institute, Cumming School of Medicine, University of Calgary, Calgary, Alberta, Canada; 2 Department of Molecular and Cellular Biology, Harvard University, Cambridge, MA; 3 Illawarra Health and Medical Research Institute, University of Wollongong, Wollongong, New South Wales, Australia; 4 Department of Neuroscience, University of Texas at Austin, Austin, TX; 5 Sechenov Institute of Evolutionary Physiology and Biochemistry, St. Petersburg, Russia; 6 Department of Biological Sciences, McMaster University, Hamilton, Ontario, Canada

## Abstract

Batrachotoxin (BTX), an alkaloid from skin secretions of dendrobatid frogs, causes paralysis and death by facilitating activation and inhibiting deactivation of eukaryotic voltage-gated sodium (Nav) channels, which underlie action potentials in nerve, muscle, and heart. A full understanding of the mechanism by which BTX modifies eukaryotic Nav gating awaits determination of high-resolution structures of functional toxin–channel complexes. Here, we investigate the action of BTX on the homotetrameric prokaryotic Nav channels NaChBac and NavSp1. By combining mutational analysis and whole-cell patch clamp with molecular and kinetic modeling, we show that BTX hinders deactivation and facilitates activation in a use-dependent fashion. Our molecular model shows the horseshoe-shaped BTX molecule bound within the open pore, forming hydrophobic H-bonds and cation-π contacts with the pore-lining helices, leaving space for partially dehydrated sodium ions to permeate through the hydrophilic inner surface of the horseshoe. We infer that bulky BTX, bound at the level of the gating-hinge residues, prevents the S6 rearrangements that are necessary for closure of the activation gate. Our results reveal general similarities to, and differences from, BTX actions on eukaryotic Nav channels, whose major subunit is a single polypeptide formed by four concatenated, homologous, nonidentical domains that form a pseudosymmetric pore. Our determination of the mechanism by which BTX activates homotetrameric voltage-gated channels reveals further similarities between eukaryotic and prokaryotic Nav channels and emphasizes the tractability of bacterial Nav channels as models of voltage-dependent ion channel gating. The results contribute toward a deeper, atomic-level understanding of use-dependent natural and synthetic Nav channel agonists and antagonists, despite their overlapping binding motifs on the channel proteins.

## Abstract (precis)

Batrachotoxin (BTX) causes paralysis and death by activating “pseudosymmetric” eukaryotic sodium (Nav) channels. Finol-Urdaneta et al. investigate its action on the prokaryotic homotetrameric homologues NaChBac and NavSp1, revealing use-dependent facilitation of activation, and inhibition of deactivation, caused by BTX binding to the symmetric pore.

## Introduction

**Figure 1.**
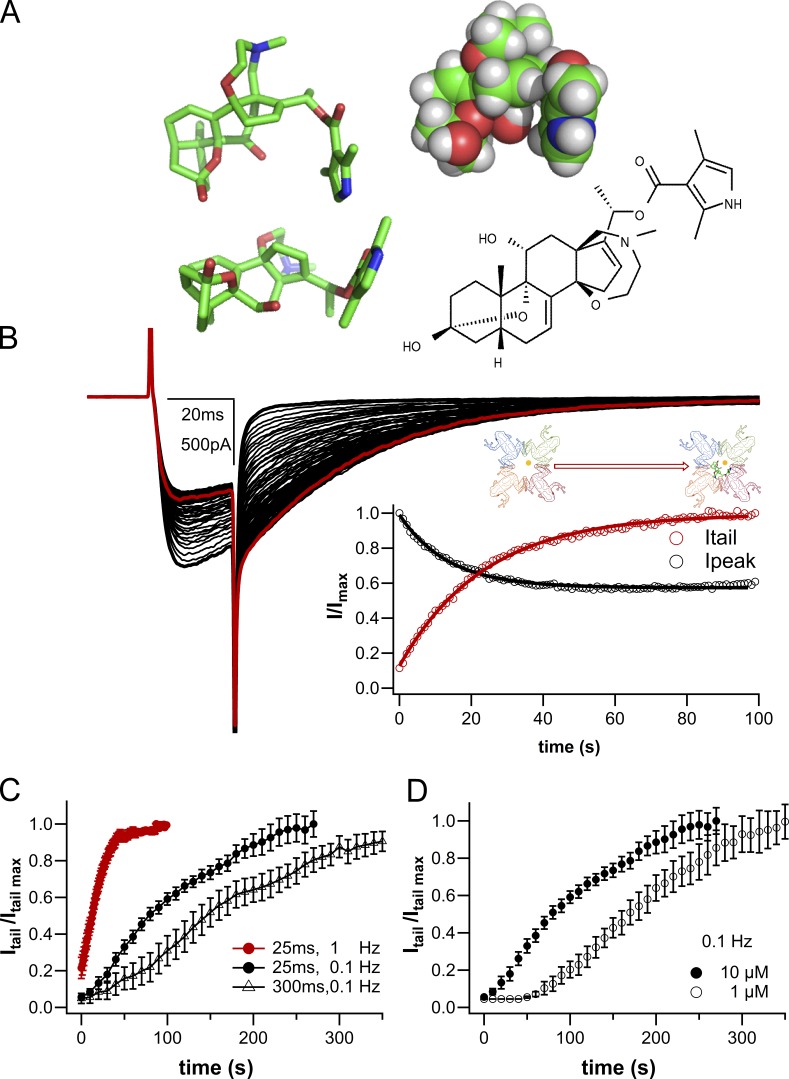
**NaChBac modification by BTX. (A)** Different representations of the BTX structure. Note the close apposition of the oxygen triad and the pyrrole ring. Electron lone pairs of the oxygen atoms and π electrons in the pyrrole ring collectively attract a metal ion, which would experience only minimal repulsion from the protonated nitrogen in the seven-member ring. **(B)** Representative NaChBac whole-cell current traces during BTX modification at 1-Hz stimulation (*V*_h_ = −120 mV, *V*_t_ = −10 mV, 10 μM BTX 140Cs_i_/142.5Na_o_). The red trace is the 100th pulse. Inset: Diary plots for normalized *I*_peak_ (open black circles) and *I*_tail_ (open red circles). The cartoon inset of the inner pore structure is expanded in the precise molecular model of Fig. 5. **(C)** Time course of change in tail current initial amplitudes during BTX dialysis into the cell during repeated depolarizations (to −10 mV; V_h_ = −120 mV) at 0.1 Hz (pulse duration, 25 ms: black circles, *n* = 8; or 300 ms: open triangles, *n* = 6) and 1 Hz (25 ms: red circles, *n* = 6). The increase in tail current amplitude (I_tail_) was used to follow BTX modification. **(D)** The rate of channel modification by BTX depends on its concentration. An increase in [BTX] in the patch pipette leads to faster modification (1 μM: open circles, *n* = 8; 10 μM: closed circles, *n* = 7; 0.1 Hz).

More than 30 years after the publication of Boris Khodorov’s review, “Batrachotoxin as a tool to study voltage-sensitive sodium channels of excitable membranes” (Khodorov, 1985), its relevance endures. Batrachotoxin (BTX) is an excitatory component in the skin secretions of dendrobatid frogs, which advertise their lethal armament with their gaudy colors (Daly et al., 1965; Tokuyama et al., 1969; Albuquerque et al., 1971). The hydrophobic alkaloid BTX (Fig. 1 A) penetrates cell membranes and activates the voltage-gated sodium (Nav) channels of muscle, nerve, and heart. BTX modification of Nav gating is facilitated by repetitive activation with protocols running from hundreds of milliseconds to a few minutes, and BTX modification is considered to be irreversible on timescales of a few minutes (Fig. 1, B–D; also see Supplemental information, Figs. S1 and S2).

Thus, BTX radically modifies eukaryotic Nav function (Brown, 1988). Its binding shifts Nav activation curves toward negative voltages and suppresses both fast and slow inactivation to yield massive, lethal increases in excitability, ultimately causing paralysis. BTX increases channel-opening probability over a wide range of potentials (Huang et al., 1982; Krueger et al., 1983) and modifies open-channel properties to decrease both unitary conductance and ion selectivity. Under whole-cell voltage clamp, an obvious BTX-induced change in eukaryotic Nav channel gating is the complete loss of fast inactivation during repeated depolarizing pulses.

Studies that directly compare unmodified and BTX-modified channels under nearly identical ionic conditions show that single-channel conductance is reduced (Quandt and Narahashi, 1982; Shenkel et al., 1989; Correa et al., 1991). BTX-modified Nav channels remain sodium selective (Fig. S3), but discrimination among sodium and other ions is generally diminished to an extent that may depend on the particular measure of selectivity used (Catterall, 1975; Behrens et al., 1989; Correa et al., 1991) and the channel isoform studied. Single-channel studies revealed that gating of individual BTX-modified channels may also undergo repeated, sudden, spontaneous shifts in the half-activation voltage on a timescale of seconds to minutes (Moczydlowski et al., 1984; Chabala et al., 1991).

Bacterial sodium (NavBac) channels lack the characteristic “hinged-lid” fast inactivation mechanism characteristic of eukaryotic Nav channels (Pavlov et al., 2005). It has been suggested that slow inactivation mechanisms may be conserved among prokaryotic and eukaryotic Nav channels (Irie et al., 2010), even though mechanisms have not been fully established. In NavBac channels, BTX modification is displayed as an increase in tail current amplitudes during repeated stimulation (Fig. 1 B), as is also evident in eukaryotic channels (Wang et al., 2001; Li et al., 2002).

Structures of eukaryotic Nav channels have recently become available (Shen et al., 2017, 2018; Yan et al., 2017), but it is unknown whether these represent functionally relevant Nav channel states. Nonetheless, the tractability of NavBac channels to coupled structural and functional study is clear (Payandeh et al., 2012; Shaya et al., 2014; Payandeh and Minor, 2015; Ahern et al., 2016; Arrigoni et al., 2016; O’Reilly et al., 2017; Sula et al., 2017; Chatterjee et al., 2018) and thus underlines the importance of prokaryotic Nav channels in the overall understanding of sodium channel function. Here, we have explored BTX actions in NaChBac and NavSp1, prokaryotic Nav channels for which much relevant structural information is available. Furthermore, we began exploring the interactions of BTX and lidocaine, which bind to overlapping channel sites within NavBac channels, thus contributing to the understanding of local anesthetic actions in both prokaryotic (Lee et al., 2012a,b; Corry et al., 2014; Shimomura et al., 2016) and eukaryotic channels (Noebels, 2002; Catterall and Swanson, 2015; Ahern et al., 2016).

To investigate the structural basis of our observations on BTX action, we built a homology model of NaChBac based on the x-ray structure of the open sodium channel NavMs (Sula et al., 2017), as described previously (Bruhova and Zhorov, 2010). The model of the BTX-bound NaChBac complex incorporates major characteristics seen in the earlier model of a BTX-bound insect Nav channel (Du et al., 2011).

Despite some differences in phenomenology, our observations on BTX modification of two functionally distinct bacterial Nav channels, NaChBac and NavSp1, reflect the fundamental mechanism by which BTX modifies both pro- and eukaryotic Nav channels. We suggest that BTX interacts with an overlapping set of homologous residues present in pore domains of both families of Nav channels. We thus identify a structurally accessible system that suggests broadly shared features of Nav channel gating and points toward both direct and allosteric actions of BTX and related, use-dependent channel modulators.

## Materials and methods

### Mutagenesis

The original construct for the *Bacillus halodurans* Na^+^ channel (NaChBac, or NavBh) in pTracer-CMV2 (Invitrogen) was provided by D. Clapham (Howard Hughes Medical Institute, Children’s Hospital, and Harvard University, Boston, MA). Sp1 from *Silicibacter pomeroyi* was obtained from D. Minor (Cardiovascular Research Institute, University of California, San Francisco, San Francisco, CA). Single amino acid mutants were generated using overlapping PCR amplification with oligonucleotides baring the sequence for the desired amino acid substitutions. All clones were completely sequenced.

### Electrophysiology

Mammalian TSA201 cells (Margolskee et al., 1993) were transfected with wild-type and mutant channel cDNAs with Lipofectamine 2000 (Fisher Scientific). Whole-cell patch-clamp recordings were made at room temperature (20–22°C) with an Axopatch 200B amplifier (Molecular Devices). Patch pipettes were pulled from Corning 8161 glass (Potash-Rubium-Lead, softening temperature, 600°C, dielectric constant, 8.3; Harvard Apparatus) to a resistance of 2–3.5 MΩ. Recordings were made 18–24 h after transfection in control (CTR) external solution that contained (in mM) 142.5 NaCl, 2 CaCl_2_, 2 MgCl_2_, 10 glucose, and 10 HEPES, pH 7.4. Intracellular (pipette) solutions were adjusted to pH 7.3 with CsOH and contained (in mM) 105 CsF, 35 CsCl, 10 EGTA, 10 HEPES, and 10 glucose. In the main text, solutions are identified by specifying only the major monovalent cations (e.g., 140 Cs_i_/142.5 Na_o_); minor components were as for the CTR external and internal solutions in the previous sentence.

BTX (0.5 mM stock solution in ethanol) was a generous gift of John Daly (National Institutes of Health, Bethesda, MD). BTX was diluted in internal solution to the desired concentration (1–10 µM) and applied through the recording pipette.

Only cells expressing peak sodium currents between 1 and 5 nA were used to ensure good current resolution while maintaining adequate voltage control. Series resistance compensation was applied conservatively to favor voltage-clamp stability and was typically 50–60%.

### Data analysis

Data were analyzed using Clampfit (Molecular Devices) and Igor (WaveMetrics) software. Peak I-V curves were fitted using Eq. 1:

$$I(V)=(V-V_{\mathrm{rev}})*G_{\max}/\left[1+\exp\left(\frac{V_{0.5}-V}{V_{\mathrm{slope}}}\right)\right],$$ (1)

where *I* is the macroscopic current, *V* is the command potential, *V*_rev_ is the reversal potential (mV), *G*_max_ is the maximal conductance (*S*), *V*_0.5_ is the half-activation potential (mV), and *V*_slope_ is the slope factor (mV/e-fold).

Initially, for comparison with published work on both pro- and eukaryotic Nav channels, activation curves over moderate voltage ranges, as normalized *G*–*V* curves, were fit to a single Boltzmann relation of the following form:

$$\frac{G(V)}{G_{\max}}=fmc/\left[1+\exp\left(\frac{V_{0.5}-V}{\frac{RT}{z*F}}\right)\right].$$ (2)

This simple analysis was used for basic comparisons in Fig. 6 B.

**Figure 2.**
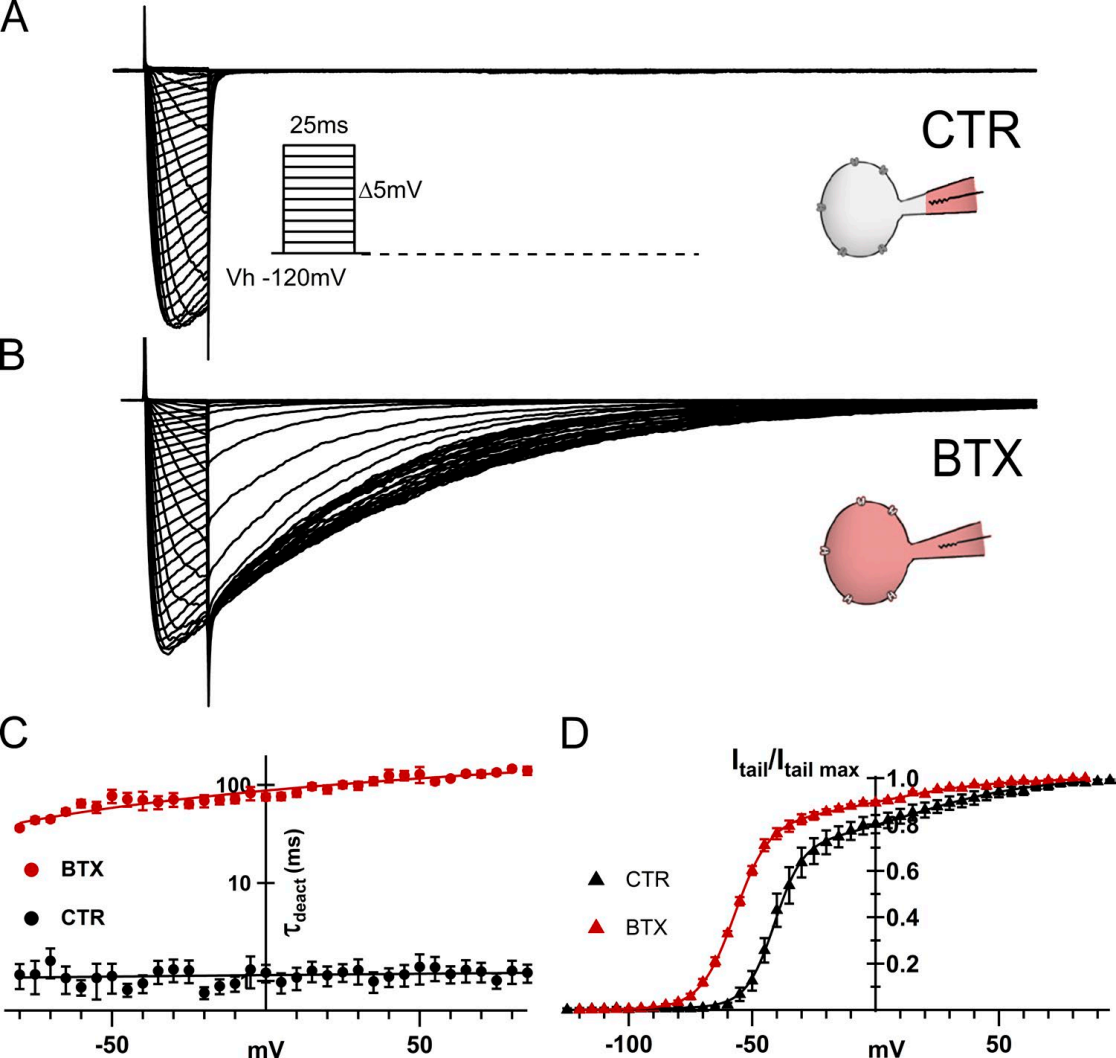
**BTX delays deactivation and shifts NaChBac activation toward more negative voltages. (A)** Representative whole-cell NaChBac currents in CTR conditions (140Cs_i_/142.5Na_o_; *V*_h_ = −120mV; *V*_t_ = −120 to +85 mV, Δ +5 mV, 0.1 Hz) from 25-ms-long pulses. Inset: Stimulation protocol. **(B)** NaChBac currents after 15-min dialysis with 10 μM BTX in the pipette solution (currents were scaled to maximal current during *I*–*V* protocol for comparison). The prominent, slowly decaying tail current at the end of each depolarizing prepulse reflects the activation facilitation by BTX. **(C)** Deactivation time constants (τ_deact_) in control (closed black circles) and after BTX modification (closed red circles). τ_deact_ from exponential fits to the tail current decay upon return to *V*_h_ after 25-ms conditioning pulses (*n* = 6 per condition). **(D)** Activation curves (normalized to *I*_tailmax_ at 90 mV) from the envelope of initial tail current amplitudes after 25-ms depolarization from *V*_h_ −120 mV as in *I*–*V* pulse protocol from A and B (BTX, closed red triangles; CTR, closed black triangles; *n* = 10 per condition).

For a more detailed analysis of NaChBac activation, over a somewhat wider range of voltage, activation curves were determined from the envelopes of tail currents and fit to a sum of two exponentials (Fig. 2 D). In this case, Eq. 2 generalizes to

$$\begin{array}{l} G(V)/G_{\max}=fmc/\left[1+\exp\left(\frac{V_{0.5}1-V}{\frac{RT}{z1*F}}\right)\right]+\\ (1-fmc)/\left[1+\exp\left(\frac{V_{0.5}2-V}{\frac{RT}{z2*F}}\right)\right], \end{array}$$ (3)

where *fmc* is the fraction of maximal conductance observed for the first or only subpopulation of channels; (1 − *fmc)* is the fraction of the second subpopulation activated (conducting) channels**;** *z*1, *z*2 are the apparent valences (gating charge) for the two-channel subpopulations, respectively; *V*_0.5_1, *V*_0.5_2 are the half-activation potentials for those respective subpopulations; *R* is the gas constant (8.314 J K^−1 ·^ mol^−1^); *T* is the temperature in degrees Kelvin [(273 + temperature in degrees Celsius) = 294]; and *F* is Faraday’s constant (9.649 × 10^4^ coulomb-mol^−1^).

The two-component “sum of Boltzmanns” analysis has precedent in an elegant study (Shyng and Nichols, 1997) in which different components of voltage-dependent spermine block were identified in a mixed population of heteromeric ATP-sensitive potassium channels to provide direct functional evidence for the octameric channel structure.

All summary data are presented as means ± SEM (*n*), where *n* is the number of determinations. Statistical significance was evaluated using the unpaired Student’s *t* test; the criterion for a significant difference was taken to be P < 0.05.

### Structural modeling of BTX binding to NaChBac

Additional details of the molecular modeling are integrated into the Results.

After sequence substitution, the model was Monte Carlo minimized to find optimal conformations of the side chains that mismatch in the NavMs and NaChBac channels. Electrostatic energy was calculated using a distance- and environment-dependent dielectric function (Garden and Zhorov, 2010). The BTX molecule was initially placed in the model according to an earlier proposal (Du et al., 2011). Two sodium ions were initially placed at positions Na_I_ and Na_II_ in the outer pore, as seen in the NavMs structure. The third sodium ion was placed between position Na_III_ and putative position Na_IV_ (Tikhonov and Zhorov, 2017), where it could interact with several of the BTX oxygen atoms. To facilitate recognition of residues in homologous positions of different channels, we show their numbers in NaChBac and, in superscript bolded, relative positions in the P-loops (**^p^**) and inner helices (**^i^**; see top row in Fig. 5 A). The complex (NaChBac channel, BTX, and two sodium ions) was optimized using a two-stage protocol. At the first stage, the backbones of the channel and BTX were kept rigid, and only side chains were optimized to relax the high-energy contacts with BTX, which appeared upon the initial BTX placement. At the second stage, all degrees of freedom were optimized.

### Kinetic modeling

Kinetic Model Builder Version 2.02 (Goldschen-Ohm et al., 2014) was used to simulate macroscopic NaChBac currents from control and BTX-modified channels. A simplified kinetic scheme (Fig. 8) was used to model *I*–*V* data for pulses of two different durations: 25 ms (*V*_h_ = −120 mV, *V*_t_ = −100 to −10 mV, Δ10 mV) and 500 ms (*V*_h_ = −120 mV, *V*_t_ = −100 mV to 20 mV; Δ20 mV). Conductance for the open "O" state in control was set to 12 pS and 6.24 pS for the BTX modified channel (Figs. 1 B and S1).

In general, the time-dependent forward (α) and backward (β) transition rates are given by α(*t*) = α_o_ exp[*q V*(*t*) / (2 *k*_b_ *T*)] and β(*t*) = β_o_ exp[−*q V*(*t*)/2 (*k*_b_ *T*)], where *V*(*t*) is the applied voltage at time *t*, *T* is temperature, *k*_b_ is Boltzmann’s constant, α_o_ and β_o_ are the rates at zero applied voltage, and *q* is the net elementary charge moved within the membrane electric field for any particular transition. This formalism assumes symmetric energy barriers for each transition, i.e., that the effective charge movements associated with the individual forward and backward transitions are equal in amplitude (*q*/2). The one exception to this (see Fig. 8 and Table S2) is the open (O) to inactivated (I_1_) step; in this case, all of the charge movement is associated with the I_1_ to O step, with zero change movement for the inactivation transition O to I_1_. The zero-voltage transition rate rates, α_o_ and β_o_, and the amounts of charge, *q*, associated with each transition, are tabulated in Table S2.

### Online supplemental material

Supplemental information available online includes: Supplemental text. The Glossary summarizes various names that have been used for different prokaryotic Nav channels, their states and modulators, and terms used in our functional analysis of their modulation by batrachotoxin. Fig. S1 shows that the reduction of peak current amplitude with BTX modification under our experimental conditions is not associated with cumulative inactivation. Fig. S2 shows the cumulative inactivation of NaChBac currents at different holding potentials. Fig. S3 displays a small but significant decrease in Na/K selectivity in BTX-modified NaChBac channels. Fig. S4 shows that NaChBac mutations (N225^i20^K and N225^i20^A) render the channel nonfunctional. Table S1 shows the partitioned energy of the BTX interaction with NaChBac residues and sodium ions. Table S2 lists optimized parameters for the model shown in Fig. 8.

## Results

The two following paragraphs, together with Figs. 1 and 2, introduce some key features of use-dependent, functional modulation of NaChBac by BTX, together with basic protocols and experimental techniques used to induce BTX’s agonistic action. More detailed analysis of the underlying events follows later in the Results.

The time course of BTX modification of eukaryotic channels is most commonly monitored by following the slowing of fast inactivation decay after 1,000–2,000 depolarizations (e.g., Huang et al., 1982). With the slower intrinsic gating kinetics of prokaryotic NaChBac, we follow BTX modification as the increase in amplitude of the tail currents (*I*_tail_) upon return to the holding potential (*V*_h_ = −120 mV), after repeated depolarizing pulses to −10 mV, as in Fig. 1 B (see also Li et al., 2002). Unless otherwise specified, all experiments reported below were performed after ≥5-min conditioning with 25-ms depolarizing pulses at 1 Hz from *V*_h_ = −120 mV and with 10 µM BTX in the patch pipette to attain a comparable level of channel modification in all cells.

### BTX modulation of NaChBac: Dependence on [BTX] and repetitive activation

The BTX structure (Fig. 1 A) fits snugly in the lumen of open eukaryotic Nav channels (Du et al., 2011) within the inner-pore region adjacent to the selectivity filter while still allowing the passage of sodium ions as represented in the cartoon inset of Fig. 1 B. BTX modification of homotetrameric prokaryotic Nav channels causes a dramatic increase in tail current amplitude and a modest but significant decrease in peak current amplitude (Fig. 1 B, diary plot inset; red open circles, *I*_tail_; black open circles, *I*_peak_) that mirrors the change in *I*_tail_ amplitude. Both the increase in *I*_tail_ and the decrease in NaChBac’s *I*_peak_ resemble actions of BTX on eukaryotic Nav channels.

With 10 µM BTX in the pipette, increasing the rate of stimulation from 0.1 to 1 Hz radically speeds up modification of the channels, as seen in Fig. 1 C. The apparent rate of modification (τ-mod) of NaChBac-mediated sodium currents was estimated by using a single exponential to approximate the time course of the *I*_tail_ increase during BTX modification. Thus, for a pulse duration of 25 ms at 0.1 Hz (black filled circles, Fig. 1 C), τ-mod is ∼92 s, whereas the same (25 ms) pulses applied at 1 Hz (red filled circles, Fig. 1 C) achieve maximal modification with a τ-mod of ∼20 s, congruent with preferential binding of BTX to an activated and/or open conformation underlying functional modification. Note that the rate of modification of NaChBac channels by 10 µM BTX is actually slower when longer (300-ms) conditioning pulses are applied (open triangles, Fig. 1 C) resulting in an apparent τ-mod of 162 s. This finding is consistent with a lower probability of modification during longer activating pulses, in which the occupancy of the inactivated state reached higher levels than during the shorter 25-ms depolarizing pulse regimen. Furthermore, a 10-fold decrease in [BTX] present in the pipette (Fig. 1 D) leads to an apparent delay of current modification reflecting slower dialysis of BTX into the cell and, as a consequence, a lag reaching its site of action within the prokaryotic Nav (see also Fig. 2). Clearly, pulse duration, frequency of stimulation, and [BTX] all impact the rate at which BTX modifies NaChBac channels.

### BTX dramatically slows deactivation and shifts activation toward negative voltages

BTX action on homotetrameric NaChBac channels is characterized by greatly enhanced tail currents and a negative shift of the voltage for 50% activation, features commonly associated with BTX binding to eukaryotic Navs. Although generally slower than eukaryotic Navs, NaChBac gating is typified by moderately rapid channel opening (activation) upon depolarization, and a very quick closing (deactivation) on return to a negative holding potential. Fig. 2 shows representative NaChBac currents before and after BTX modification. By backfilling the pipette with control (gray) intracellular solution (tip) and BTX-containing (pipette body, red in schematic insets), we recorded families of control (CTR) current traces immediately after establishing stable whole-cell configuration (before BTX diffused into the cell (Fig. 2 A) and ~15 min after BTX modification on the same cell (Fig. 2 B). We observe a striking 100-fold slowing of deactivation kinetics for the BTX-modified, NaChBac-mediated currents compared with control records obtained before BTX modification (Fig. 2 C). This dataset displays minimal voltage dependence of the deactivation time constant obtained from single exponential fits to the current decay, consistent with an open-to-closed transition that is only weakly dependent on voltage. A second striking effect is an approximately −15-mV shift in the activation of BTX-modified NaChBac currents (Fig. 2 D), as determined from instantaneous tail currents elicited after 25-ms depolarizations and plotted versus the prepulse potential. This hyperpolarizing shift qualitatively resembles those associated with BTX action on eukaryotic Nav channels (Huang et al., 1982).

A sum of two Boltzmann components better described the activation of NaChBac currents over a wide voltage range (Fig. 2 D and Eq. 3, Materials and methods). The low-voltage component (valence, *z*1; half-activation voltage, *V*_0.5_1) appears to approximate that seen in the *G*–*V* data derived from the maximal current observed during 25-ms *I*–*V* curves (e.g., dashed lines in Fig. 6 B). At more positive voltages, *I*_tail_/*I*_tail max_ increases only gradually over a wide range with an apparent mid-point, *V*_0.5_2_,_ of ∼0 mV (Fig. 2 D, Supplemental materials, and Eq. 3).

Thus, the two most prominent agonistic effects of BTX on NaChBac function are (1) a shift to more negative voltages of NaChBac activation/channel opening (Fig. 2 D) and (2) a slowing of deactivation on return to −120 mV after each activation pulse (Fig. 2 C). Each of these augments the probability of the conducting state in the presence of BTX.

### BTX speeds the inactivation decay rate but increases the residual current

**Figure 3.**
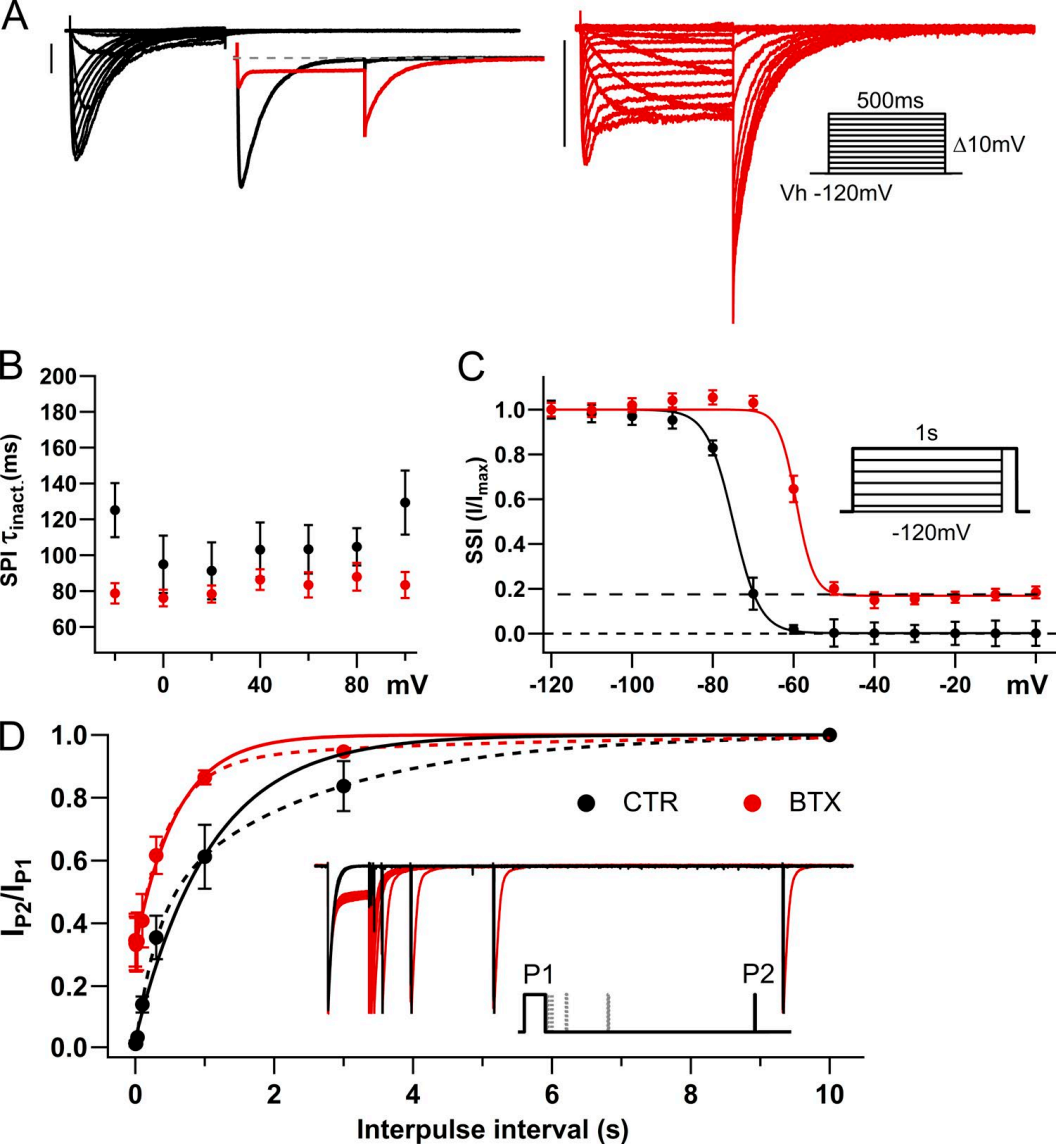
**BTX effects on NaChBac inactivation. (A)** Scaled whole-cell NaChBac currents from a 500-ms-long *I*–*V* protocol (inset on the right) in control (black), and after extracellular application of 10 μM BTX (red). Left inset: Currents at −10 ms before scaling to *I*_max_. **(B)** SPI (τ_inact_) from exponential fits to current decay during depolarization (BTX, red closed circles, *n* = 6; control, black closed circles, *n* = 6). **(C)** Steady-state inactivation plots (I/I_max_) from current at −10 mV (50 ms) after 1-s-long inactivating prepulses (inset) in control (*V*_0.5_ = −74.97 ± 2.63; black closed circles, *n* = 6) and after BTX modification (*V*_0.5_ = −59.32 ± 4.04; red closed circles, *n* = 11). **(D)** Recovery from inactivation in control and after BTX modification (*n* = 7 per condition). Solid lines represent single exponential and dashed lines double exponential fits. Inset shows NaChBac currents in CTR and after BTX modification upon a two-pulse recovery from the inactivation protocol (*V*_h_ = −120 mV; P1 = −10 mV, 1 s; interpulse 0.001, 0.003, 0.01, 0.03, 1, 3, and 10 s; P2 = −10 mV, 25 ms).

The seemingly contradictory behavior implied in this subheading is illustrated succinctly in Fig. 3 A. In the left inset, rapid inactivation decay results in a much lower peak for the BTX-modified trace, which, however, crosses the CTR trace to reach a steady state of notably larger amplitude. Single-pulse inactivation (SPI) time constants (τ_inact_) obtained from single exponential fits to the current decay during the long opening at different potentials (∼0–80 mV) show little voltage dependence. However, current decay is somewhat accelerated by BTX modification, with τ_inact_ of ∼100 ms for control conditions and ∼80 ms after BTX modification (Fig. 3 B). Steady-state inactivation of NaChBac channels, estimated from the maximal current available at −10 mV after 1-s pre-pulse depolarizations (from −120 to 0 mV in 10-mV steps), is also altered by BTX modification. The plot in Fig. 3 C shows an ∼15-mV positive shift in the inactivation *V*_0.5_ and a consistent ∼20% noninactivating fraction (residual current) from −50 to 0 mV, indicating an increase of available conductance over a wide range of voltages in BTX-modified NaChBac channels.

Compared with eukaryotic Nav channels, homotetrameric NaChBac channels display slower inactivation kinetics (Ren et al., 2001; Pavlov et al., 2005). Nevertheless, BTX can impair NaChBac inactivation in a fashion reminiscent of its effects on mammalian Nav channels, increasing the quasi–steady-state conductance at the end of moderate to long depolarizations (∼300–1,000 ms). We explored recovery from inactivation with interpulses at −120, −100, and −80 mV; however, we only detected significant recovery of NaChBac at −120 mV in control conditions within the experimentally practical time course. In this case, BTX-modified NaChBac channels recover more rapidly from inactivation (τ_rec_: 602 ± 7.1 ms, *n* = 10) than unmodified CTR channels (τ_rec_: 1,075 ± 8 ms, *n* = 8; Fig. 3 D). Overall, the effects of BTX on prokaryotic NaChBac channels and eukaryotic Nav channels are qualitatively similar. As a consequence, the channel’s kinetics are biased toward activation and persistent conduction, which can result in catastrophic sodium accumulation in the intracellular milieu.

**Figure 4.**
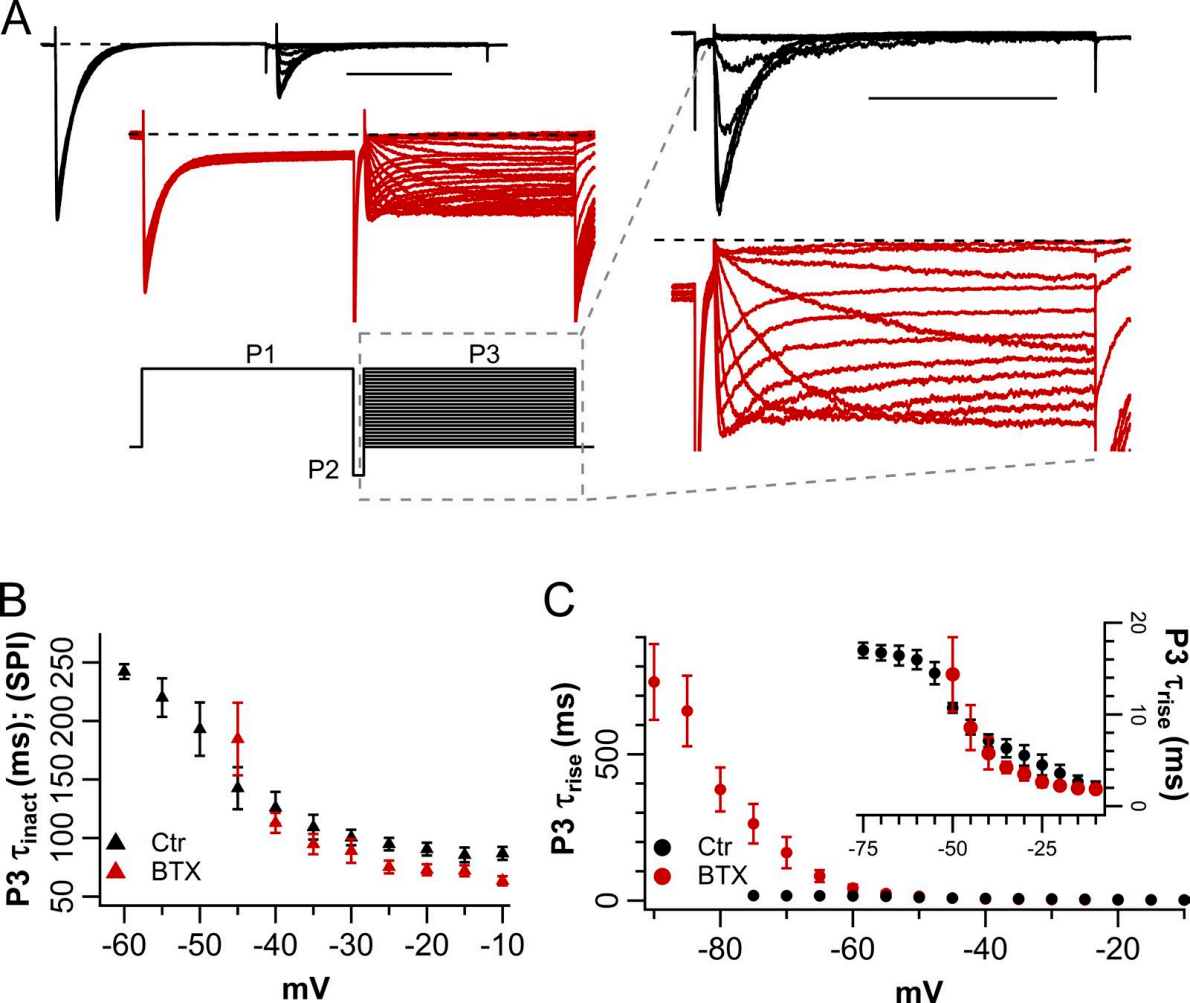
**BTX speeds recovery from inactivation and slows NaChBac reactivation. (A)** Left: Representative families of NaChBac currents in response to the three-pulse protocol shown in the inset (P1 = −10 mV, 1 s; P2 = −160 mV, 50 ms; P3 = −120 to −10 Δ +5 mV, 1 s; *V*_h_ = −120 mV; BTX, red; CTR, black). Right: Expanded P3. Scale bars, 500 ms. **(B)** SPI time constant (τ_inact_) from single exponential fits to the current decay during P3 (BTX, red triangles; CTR, black triangles). **(C)** Rise time constants (τ_rise_) obtained from single exponential fit to the early current rise (P3) observed after a brief hyperpolarizing pulse to −160 mV (P2; BTX, red circles; CTR, black circles). Inset: Expanded τ_rise_ scale (*n* = 4 per condition).

An alternate perspective on the effects of BTX comes from its action on reactivation kinetics following inactivation. We achieved this by interposing a brief, strongly hyperpolarizing pulse (P2, −160 mV; 50 ms), between a constant conditioning pulse (P1, −10 mV; 1 s) and a subsequent, variable test pulse (P3, −120 to −10 mV, Δ*V* = 5 mV; 1 s; Fig. 4), to reveal the voltage dependence of the channel’s reopening kinetics. This protocol drives fast deactivation during P2, with little recovery from inactivation, then traces the time course of reactivation during P3 from a closed-but-available to an open state.

Fig. 4 A contains representative traces of NaChBac-mediated currents in control (black), and after BTX modification (red), elicited by such a pulse protocol. Activation (τ_rise_) and inactivation (τ_inact_) time constants were estimated from fits of the product of two exponentials to the current wave forms during P3. This analysis highlights the different effects of BTX on the time courses of activation and inactivation, estimated on the same trace. The inactivation time constant, τ_inact_, provides an approximate inverse measure of the inactivation rate (SPI) during the reactivating pulse P3. Fig. 4 B shows that BTX has little effect on τ_inact_ during P3 in the range −45 to −10 mV, whereas the reactivation rate (1/τ_rise_) during P3 is slowed by BTX (Fig. 4 C; note the larger scale on the left ordinate than that in the inset). With a −120-mV holding potential, reactivation is slowed by BTX for activating voltages, −100 mV < P3 < −60 mV. The complex voltage dependence of the activation/reactivation kinetics suggests at least two classes of channel conformations, which can be rapidly repopulated when channels deactivate and/or recover from inactivation during the brief hyperpolarization in P2. This is consistent with the overall complexities of kinetics and voltage dependence of inactivation, illustrated in Figs. 3 and 4.

### Molecular bases of BTX actions: Binding to homotetrameric NavBac channels

**Figure 5.**
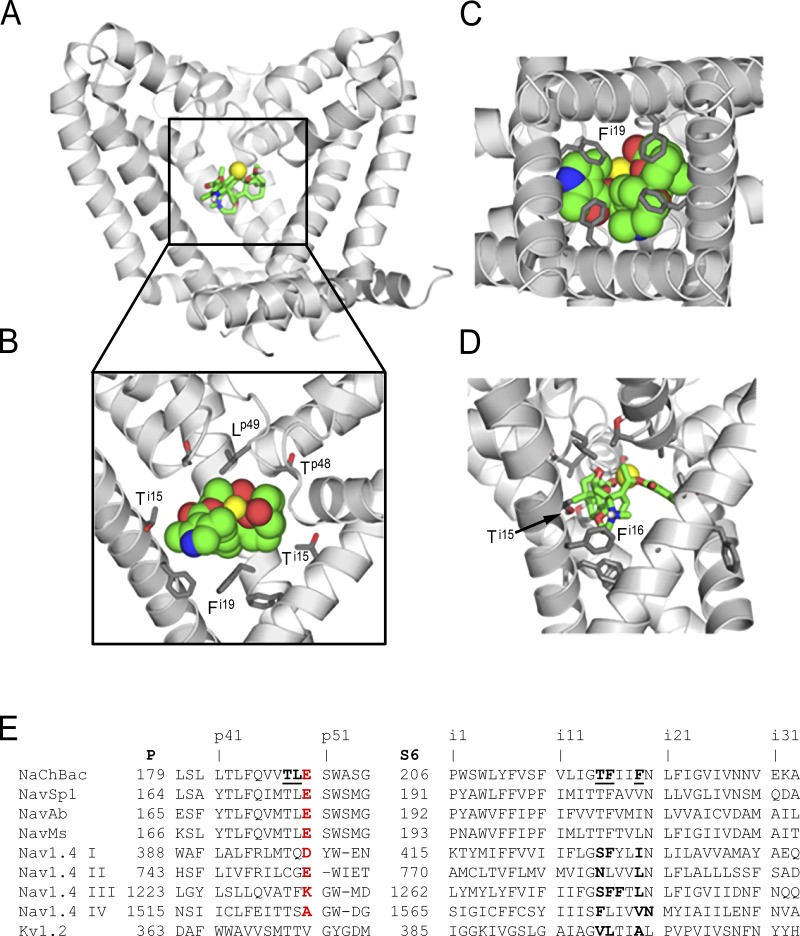
**Molecular structural basis for NaChBac modification by BTX.** The NaChBac channel homology model with BTX and a sodium ion (yellow). The model was built using the x-ray structure of the open sodium channel NavMs. Sodium ions can pass through the BTX-bound channel. **(A)** Side view of the pore domain. **(B)** Enlarged view of the BTX binding site. The front subunit is removed for clarity. BTX fits snugly in the inner pore below the P1 helices. Pore-facing residues (Thr220^i15^ and Phe224^i19^) contribute to the BTX receptor. **(C)** View from the cytoplasm. **(D)** The charged ammonium group of BTX is engaged in cation-π interaction with Phe221^i16^. The arrow indicates the location of the Ti15 sidechain (see also B). **(E)** Sequence alignment of P-loops and S6 helices in sodium and potassium channel Kv1.2 (KCNA2_HUMAN). NaChBac residues that interact with BTX in our model are underlined. Residues critical to BTX modification in Nav1.4 are bolded. Even if BTX could reach the open pore of the Kv1.2 channel, residues Val399^i15^, Leu400^i16^, and Ala403^i19^ would not attract BTX as strongly as do polar (Thr220^i15^) and aromatic (Phe221^i16^ and Phe224^i19^) residues in homologous positions of NaChBac.

Previous models of BTX binding to eukaryotic sodium channels were built from x-ray crystals of open potassium channels (Jiang et al., 2003; Long et al., 2005). For modeling of BTX binding to open NaChBac, we have used the x-ray structure of the open NavMs channel (Sula et al., 2017) as template. The present model (Fig. 5, A–D) has the same general folding as Kv channels, but the pore is wider in the region where BTX binds at the level of Thr220^i15^ and, correspondingly, the interfaces between S6 helices are expanded. Fig. 5 E includes residue labels that are universal for P-loop channels, which appear superscripted henceforth. Additional details on homology modeling with the ZMM program can be found in earlier work (e.g., Bruhova and Zhorov, 2010) and the Materials and methods (see Supplemental materials).

The final complex (Fig. 5) is congruent with an earlier model of BTX binding to the eukaryotic NavBg channel (Du et al., 2011). We show that despite NaChBac’s nominal fourfold symmetry, asymmetric BTX interacts differently with individual subunits. We designate subunits A–D as repeats I–IV in eukaryotic channels. The list of main contributions to the BTX interaction energy is summarized in Table S1. BTX is horseshoe shaped (Fig. 1 A) with a predominantly hydrophobic external face that effectively interacts with the rings of Thr220**^i15^**, Phe221**^i16^**, and Phe224**^i19^** residues. The notable exceptions are Thr220**^i15^** and Phe220**^i16^** in subunit “C” (Fig. 5 E). The latter provides a cation-π contact with the charged amino group of BTX, whereas the Thr220**^i15^** residue forms an H-bond with BTX. The “oxygen triad” of BTX (Kosower, 1983) interacts with the sodium ion in a manner that resembles interactions of water molecules with Na^+^. Due to the wide opening at the level of BTX binding, a “BTX-bound Na^+^” ion is able to occupy a position near the pore axis. Thus, despite obvious differences between eukaryotic Nav channels and NaChBac, the key determinants of BTX binding seem well conserved among them.

### The BTX-binding site is conserved among NavBac channels and Nav1s

**Figure 6.**
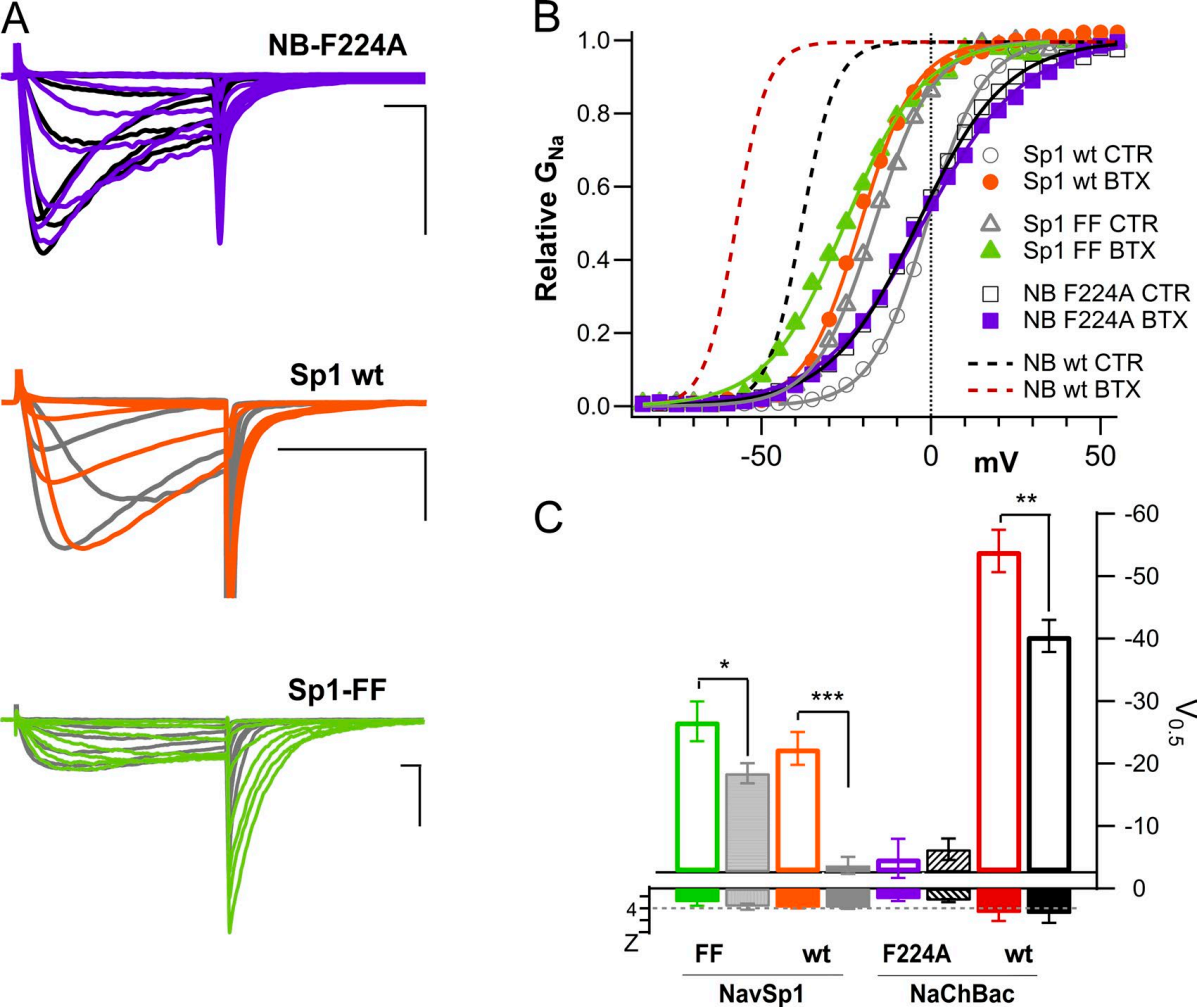
**BTX modification of NaChBac F224A; NavSp1 and NavSp1 FF mutant. (A)** Representative whole-cell currents in the presence of 10 μM BTX (*V*_h_ = −120 mV, 0.1 Hz). Top: NaChBac NB F224A (BTX, purple; CTR, black). Middle: Sp1 wt (BTX: orange; CTR, gray). Bottom: Sp1 V209F-L212F (BTX, green; CTR, gray). Scale bars, 10 ms, 1 nA. NB, NaChBac. **(B)** Relative *G*–*V* plots in control and after BTX modification for Sp1 wt (BTX, filled orange circles; control, open circles), Sp1 FF (BTX, filled green triangles; control, open triangles), and NB-F224A (BTX, filled purple squares; control, open squares). Dotted lines from NaChBac wt (BTX, red; CTR, black) activation are included for reference. **(C)** Half activation potentials (V_0.5_, right axis) and slopes (Z, left axis) from Boltzmann fits to the relative conductance plots in B. Currents were normalized to maximal peak current within the *I*–*V*. Data comparisons: *, P < 0.05; ***, P < 0.001.

In our experiments, NavBac residues, whose mutation altered the susceptibility to modification by BTX (F224**^i19^**A; Fig. 6), and/or eliminated expression of functional channels (N225^i20^K; Fig. S4), aligned with those found to affect BTX action in eukaryotic Nav channels (Wang et al., 2001). The NaChBac mutant F224^i19^A showed faster inactivation kinetics (Fig. 6 A) and a positive shift in activation *V*_0.5_ (Fig. 6, B and C) in control compared with wild-type NaChBac channels. Upon BTX application, F224A displayed a modest increase in tail current amplitudes, little change in deactivation kinetics, and no detectable shift of activation voltage dependence. Therefore, residue F224**^i19^** represents a critical component for the agonistic actions of BTX on NaChBac-mediated currents.

We also explored BTX modification of the homologous prokaryotic sodium channel from *Silicibacter pomeroyi*, NavSp1. This BacNav has significantly faster gating kinetics than NaChBac. BTX modified NavSp1 kinetics and voltage dependence similarly to those of NaChBac and eukaryotic Nav channels. That is, BTX dialysis of NavSp1-expressing cells showed prolonged tail currents (Fig. 6 A; middle panel, CTR: gray; BTX: orange), and activation was left shifted by −20 mV (Fig. 6, B and C). Moreover, NavSp1 currents with significantly slower kinetics were generated by homologous substitution of phenylalanine residues that affected BTX modification in eukaryotic channels (Wang et al., 2006) and in NaChBac (current work). The NaChBac-like Sp1-FF double mutant V209**^i19^**F/L212**^i22^**F displays massive tail currents upon BTX modification (Fig. 6 A, bottom [BTX, green; CTR, gray]) with a concomitant leftward shift in activation *V*_0.5_ (Fig. 6, B and C). Furthermore, as with NaChBac, modification rates of NavSp1-FF were accelerated by increasing the frequency of the conditioning pulses (0.2–2 Hz).

Mutation of asparagine N225**^i20^** to alanine or arginine in NaChBac yielded nonfunctional channels (no detectable sodium currents upon transient transfection after 24–72 h; Fig. S4). Given the homotetrameric nature of prokaryotic sodium channels, we hypothesize that NaChBac mutants with substitutions of N225**^i20^** resulted in nonfunctional channels because alanine residues in this position in all four monomers cannot support the critical open-state H-bonds (N**^i20^**–N**^i29^** proposed by Tikhonov et al., 2015). This mutation would dramatically destabilize the open state of homotetrameric NavBac channels. For the well-studied Nav1.4 mutant N**^i20^**A, function is likely retained because only one of the four interrepeat open-state H-bonds is broken. Modulation of inactivation kinetics and steady-state channel availability are likely to be allosteric corollaries of the direct effects of BTX on channel activation/deactivation transitions. However, we cannot discard the possibility that channels were not translated or trafficked to the plasma membrane, despite the absence of signs of ER stress due to orthologous protein accumulation (Sano and Reed, 2013).

These observations reinforce the following ideas: (1) the primary functional change induced by BTX is stabilization of the open state, likely by changing the voltage dependence of channel activation/deactivation, and (2) a reduced degree of inactivation during the conditioning pulses favors channel modification (e.g., Sp1-FF; Fig. 6). The latter observation is consistent with the results shown in Fig. 1 C, in which 300-ms pulse trains were less effective at inducing BTX modification than 25-ms pulses of the same amplitude and frequency. The proposed binding mode of BTX (Fig. 5) is consistent with our experimental results and is similar to that for the cockroach sodium channel BgNa_V_ (Du et al., 2011).

Among natural channels and experimental constructs, the possibility of several subtly differing conformations arises. These could underlie the observed complexities of inactivation kinetics and its voltage dependence (Figs. 3 and 4). Similarly, a group of related functional states may allow the relatively simple kinetic model (Fig. 8) to simulate the kinetic changes that result from BTX binding.

### Lidocaine- and BTX-binding sites overlap in NavBac channels

**Figure 7.**
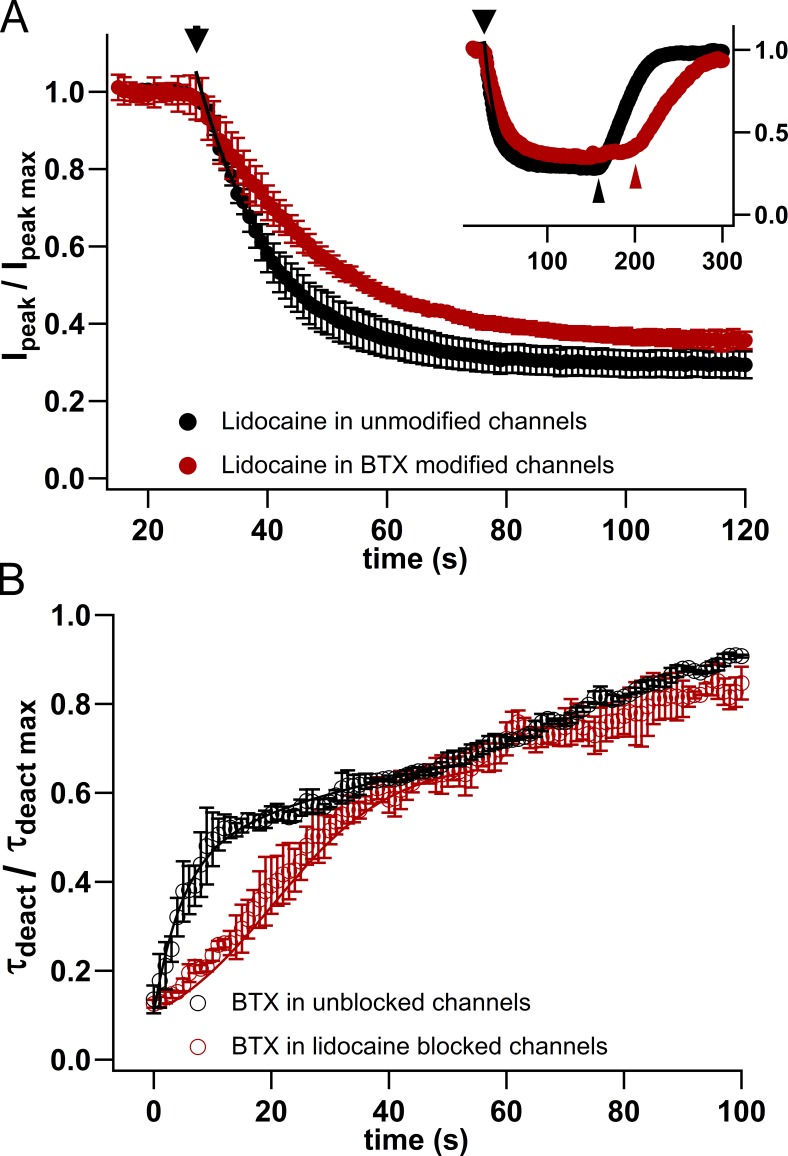
**BTX/lidocaine interactions. (A)** Inhibition of NB-mediated currents by lidocaine in unmodified (filled black circles) and 10 μM BTX–modified (filled red circles) NaChBac channels. Average diary plot of three experiments where 500 μM lidocaine blocked Na^+^ currents through unmodified and BTX-treated NaChBac channels. Main panel (*washin*) and insert show normalized peak current versus time. Thick arrowheads indicate the beginning of *washin*, and thin arrowheads indicate the beginning of *washout* (unmodified τ_wi_: 13.56 ± 1.14 s; BTX modified τ_wi_: 22.72 ± 1.97 s; *n* = 3 per condition). **(B)** BTX modification was followed in the presence of 1 mM lidocaine. Deactivating NaChBac currents were fitted to a double exponential (in order to account for control, lidocaine, and BTX within the same protocol). The BTX -sensitive time constant (>3 ms) was normalized to its maximal value and plotted against time. Lidocaine significantly delays the early phase of BTX modification (CTR τ_wi_: 8 ± 0.48 s; lidocaine τ_wi_: 55 ± 9 s; *V*_h_ = −120 mV, *V*_t_ = −10 mV, 1 Hz; *n* = 3 per condition).

In eukaryotic channels, the binding sites for lidocaine and BTX are thought to overlap, suggesting that the binding of one ligand would interfere with subsequent binding of the second. Therefore, we performed coapplication experiments in which BTX-modified NaChBac currents were exposed to the local anesthetic, lidocaine, or treated with BTX after equilibration in the presence of lidocaine (Fig. 7). Our experiments showed that both block and unblock by lidocaine were slower for BTX-treated channels than for nonmodified channels (Fig. 7 A), suggesting that once BTX has bound within NaChBac’s inner cavity, it hinders lidocaine’s access to, and escape from, its binding site. When the order of application was reversed, BTX modification in the presence of lidocaine was slower than in controls (Fig. 7 B). Thus, as proposed for eukaryotic channels, our results are consistent with the presence of overlapping binding sites for local anesthetics and BTX within the NaChBac inner cavity.

## Discussion

The first description of the functional expression of a prokaryotic sodium channel (Ren et al., 2001) contained several surprises: (1) NaChBac emerged as a voltage-gated, sodium-selective channel, despite an earlier suggestion that the gene encoded a voltage-gated calcium channel, based on a selectivity filter lined by four glutamate residues (Durell and Guy, 2001); (2) NaChBac was insensitive to the iconic Nav-channel pore blocker, tetrodotoxin; (3) NaChBac was blocked by established Cav blockers (Ni^2+^, Cd^2+^, dihydropyridines, and mibefradil); and (4) NaChBac showed slower kinetics than eukaryotic Nav channels but generated depolarization-induced, inactivating inward currents, generally similar to channels in nerve and muscle, despite lacking an obvious hinged-lid inactivation gate within its sequence.

NaChBac’s observed crossover pharmacological profile prompts a number of questions, which, together with our own results (Figs. 1, 2, 3, 4, and 6), provoke emerging hypotheses regarding the subtleties of mechanism shown by BTX’s agonistic action on NaChBac, the determinants of BTX targeting, and possible factors driving its evolution. We discuss these issues below.

BTX’s dramatic agonistic action on NaChBac and NavSp1 precludes a rigid requirement for four distinct domains, like those of the eukaryotic Nav1 channels, for its action. Nonetheless, a role for subtle structural asymmetry in the pore remains possible, as suggested by asymmetry in crystal structures of NavAb. This asymmetry and reports of two likely inactivated structures hint at novel possibilities for drug development (Payandeh et al., 2012). We argue that BTX binds in a favored orientation in NaChBac (Fig. 5).

Highly conserved S6 asparagines (N^i20^s) are close to BTX-binding residues, but they face away from the pore lumen and therefore do not form direct contacts with BTX in our model. The asparagines, N^i20^, are proposed to stabilize the open pore by forming H-bonds with polar residues at the C-ends of neighboring S6s (Tikhonov et al., 2015; Du et al., 2018). Thus, the N^i20^s of each monomer or domain help to keep the access pathway open for BTX from the cytoplasm to the full array of amino acids that contribute to its binding to the activated states.

### Simulating the kinetics of BTX-modified NaChBac channels

**Figure 8.**
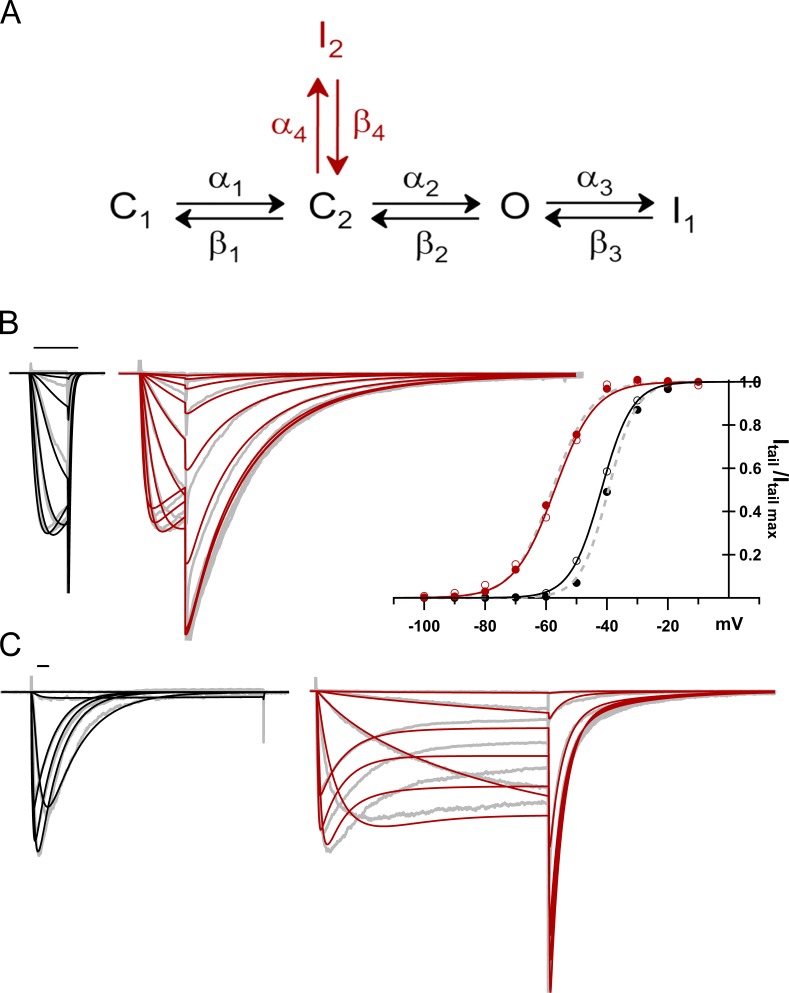
**Kinetic scheme of NaChBac in control and after BTX modification. (A)** State diagram used for simulations. Black lettering corresponds to states and transitions common to control and BTX modified currents, whereas red lettering denotes the I2 state and connecting transitions used only for the BTX simulations. **(B)** Left: Superimposed reference (gray) and simulated current traces (BTX, red; CTR, black) traces from 25-ms *I*–*V* stimulation (−100 to −10 mV). Right: Activation plots from experimental (BTX, red closed circles; CTR, black closed circles; gray dotted lines are single-Boltzmann fits) and simulated currents (BTX, red open circles; CTR, black open circles; solid lines are single Boltzmann fits). **(C)** 500-ms *I*–*V* reference and simulated current traces (−100 to 20 mV, colors as in B). Scale bars, 25 ms.

The simplified kinetic model in Fig. 8 approximates quite complex changes in the gating kinetics of NaChBac induced by BTX. A major generalization in the model involves the reduction of the activation pathway to two closed states. Despite its simplicity, this model accounts for the qualitative kinetic features of our data and the canonical hyperpolarizing shift in activation induced by BTX. On our timescales, we do not resolve any obvious sigmoidicity during activation, so additional closed states were not required to generate that feature, in contrast to the iconic Hodgkin–Huxley model (Hodgkin and Huxley, 1952) and its adaptation in a study of NaChBac (Kuzmenkin et al., 2004). In the activating voltage range, the immediate preopen state, C2, is occupied by BTX. To accommodate changes in the kinetics and voltage dependence of inactivation upon BTX modification, we added a second inactivated state, I2, which is only occupied significantly after BTX modification. We were unable to improve the fits by adding an I2 state in any other positions of the scheme.

The following qualitative features of BTX action, from our own and published data, are mimicked in the calculations: (1) a leftward shift of voltage-dependent activation (Fig. 2 D), (2) slowing of deactivation following a voltage step back to negative voltages after an activating depolarization (Fig. 2 B; and Fig. 8, B and C), and (3) similar time courses shown by the successive decreases in peak current and increases in tail current during BTX modification by a series of activating depolarizations (Fig. 1 B), which necessarily emerge from the reduced unitary conductance assumed for the BTX-modified channel (6.24 pS vs. 12 pS for the control).

Note that here, we attempt to simulate the properties of the BTX-modified channels, but not the kinetics of NaChBac modification by BTX. On the timescale of the reference current traces simulated, the model reasonably recapitulates activation and allows the relatively slow transitions within the BTX-modified channel to be simulated with the inclusion of a single additional inactivated state, I2. Nonetheless, given the limited number of transitions considered, we cannot link movements of specific channel elements with particular transitions in the model. Further information regarding the calculations and parameter values used to generate the traces in Fig. 8 are provided in the Materials and methods and Table S2.

Our observations, in conjunction with other studies, are consistent with the following: (1) The key action of BTX is to indirectly modify the outcome of voltage sensor function through its allosteric coupling to the BTX-binding site in the pore domain. The model of BTX binding (Fig. 5) suggests a molecular rationale for this action at a distance: when the bulky BTX binds in the inner cavity of the pore, it hinders closure (deactivation). This would hamper the return movement of the S4–S5 linker helices and S4s into the conformations that correspond to the deactivated channel. (2) We suggest that modification of inactivation by BTX in both prokaryotic and eukaryotic Nav channels is functionally incidental to its binding within the pore rather than a direct interaction of the ligand with the voltage sensor (see also Fig. 6) and that different inactivation mechanisms may affect BTX binding differently. Wide-ranging studies by S.-Y. and G.K. Wang underline the rich complexity and subtlety of BTX’s actions and interactions with other agents that bind at nearby or overlapping sites (Wang et al., 2007) and also show that substitution of a single asparagine residue mimics the resistance to BTX seen in the muscle channel, Nav1.4, from the poison dart frog, *Phyllobates terribili*s (Wang and Wang, 1999, 2017). (3) Inhibitory local-anesthetic–like molecules and agonists like BTX, while antithetic in their dominant functions (Fig. 7), bind in the same general location and share functional properties, such as use-dependent binding and pore occlusion (partial, in the case of BTX). Overlapping sets of key residues in NaChBac underlie these opposing actions, as indicated in the Results and other studies (e.g., Shimomura et al., 2016). The use or state dependence of their therapeutic or biological mechanisms share much in common. Ultimately, BTX acts as a NavBac activator, because its stabilization of conducting states functionally outweighs its limited blocking action. Du Bois and collaborators show clearly that small molecules based on the BTX structure can act as potent Nav inhibitors, despite their molecular heritage (Logan et al., 2016; Toma et al., 2016). Extension of these studies, including further experiments with additional use-dependent modulators (Lee et al., 2012a,b; Ahern et al., 2016), should pave the way for a deeper understanding of mechanisms of both excitatory and inhibitory modulation and may uncover new applications for use-dependent Nav-targeted ligands. The rapidly increasing availability of high-resolution structural data from prokaryotic and eukaryotic Nav channels (Shen et al., 2017, 2018), as well as concatenated constructs (Ahuja et al., 2015), will provide a fertile context for more detailed structural interpretation of the complex effects of BTX, as well as other activity-dependent ligands.

Overall, we demonstrate similarities of BTX interactions with homotetrameric prokaryotic Nav channels and their pseudotetrameric eukaryotic Nav channel cousins. BTX binds to residues lining the inner pore at a location similar to that which binds local anesthetics. Despite overlapping binding sites, these two well-known modulators of eukaryotic Nav channels display opposing effects in both eukaryotic and prokaryotic Nav channels. Our results, together with data from other studies, also raise the possibility of addressing broader issues, including mechanistic, biological, and evolutionary questions. Long before its adoption by dendrobatid frogs, might BTX have been a weapon in skirmishes among competing prokaryotes? Even if not, might they be adapted for antimicrobial applications (Clarke, 1997)? What molecular details determine the balance between agonistic (BTX) and inhibitory actions (local anesthetics), and how might these be used to design new therapeutics?

## Supplementary Material

Supplemental Materials (PDF)
