# Supplementary material for: Batrachotoxin acts as a stent to hold open homotetrameric prokaryotic voltage-gated sodium channels
Source: J Gen Physiol. 2019 Feb 4;151(2):186–99. doi: 10.1085/jgp.201812278 (PMC6363421; doi:10.1085/jgp.201812278)
Supplement: Supplemental Materials (PDF) [file JGP_201812278_sm.pdf]

Supplemental material

Finol-Urdaneta et al., https://doi.org/10.1085/jgp.201812278

Glossary of channel names

BacNav, NavBac, prokaryotic (bacterial) voltage-gated sodium channels

BTX, batrachotoxin

NaChBac/NB, voltage-gated sodium channel from Bacillus halodurans; also referred as NavBh

Sp1, voltage-gated sodium channel from Silicibacter pomeroyii (also called Rugeria pomeroyii)

Nav, voltage-gated sodium channels

Nav1.x, eukaryotic voltage-gated sodium channels (in mammals, x = 1–9, where numbers denote different isoforms, associated primarily with a specific tissue or cell type)

VGIC, voltage-gated ion channels, whose subfamilies include Cav, Kv, and Nav variants, based on their ion selectivity, and in a broader sense, any ion channel controlled in part by a voltage-sensing domain of four transmembrane segments, with the basic residues that sense the transmembrane electric field, concentrated in S4

Nav conformational states

Resting: Nonconducting states favored by negative or hyperpolarized voltages across cell membranes.

Open: Activated, conducting state normally transiently favored by membrane depolarization.

Inactivated: Nonconducting states typically favored by prolonged membrane depolarization.

Deactivated: Nonconducting states typically favored by hyperpolarization.

Nav steady-state and kinetic parameters

τ_{deact}, deactivation time constant

I_{tail}, current amplitude immediately after repolarization to V_h

$I_{tailmax}$, maximal current amplitude immediately after repolarization to V_h

SPI, the empirical description of the inactivation seen as a decay of current/conductance during a prolonged depolarizing pulse and does not necessarily imply a specific mechanism or a single inactivated state

SSI, steady-state inactivation

τ_{inact}, time constant of current decay during an activating pulse

I_{Px}, peak current at pulse x

τ_{rise}, time constant of early current development upon depolarization

I_{peak}, maximal current amplitude during an activating pulse

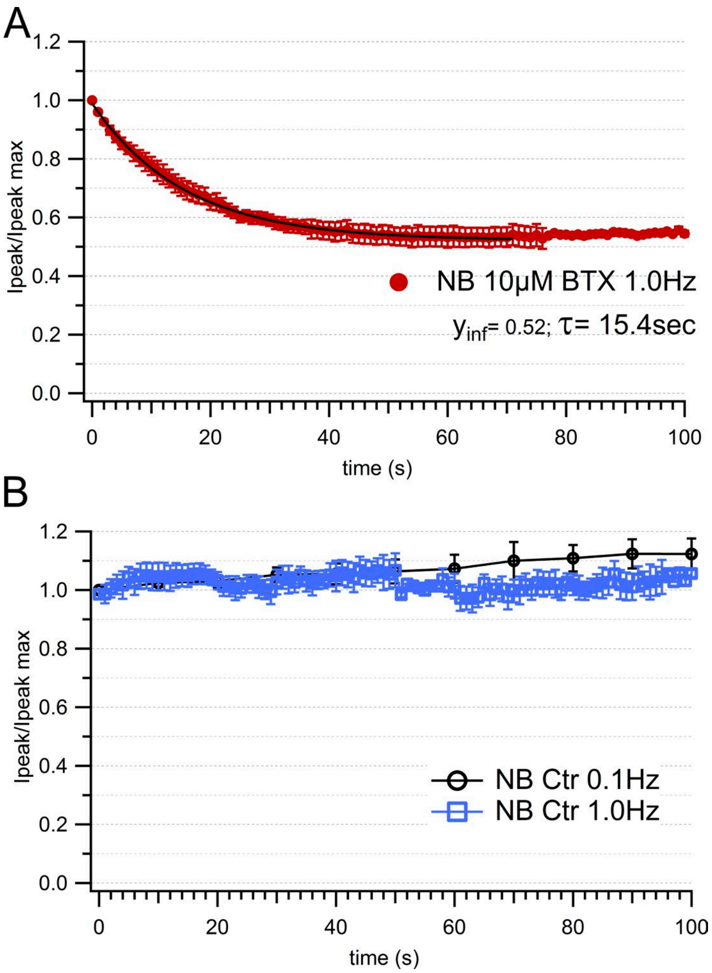

Figure S1. **The reduction of peak current amplitude with BTX modification is not associated with cumulative inactivation. (A)** Diary plot of NaChBac (NB) relative peak current during BTX modification at 1.0 Hz (*n* = 5). **(B)** Diary plot of NB relative peak current during 0.1 Hz (*n* = 4) and 1.0 Hz (*n* = 4) stimulation in control conditions. V_h = −120 mV; V_t = −10 mV; 25-ms pulses.

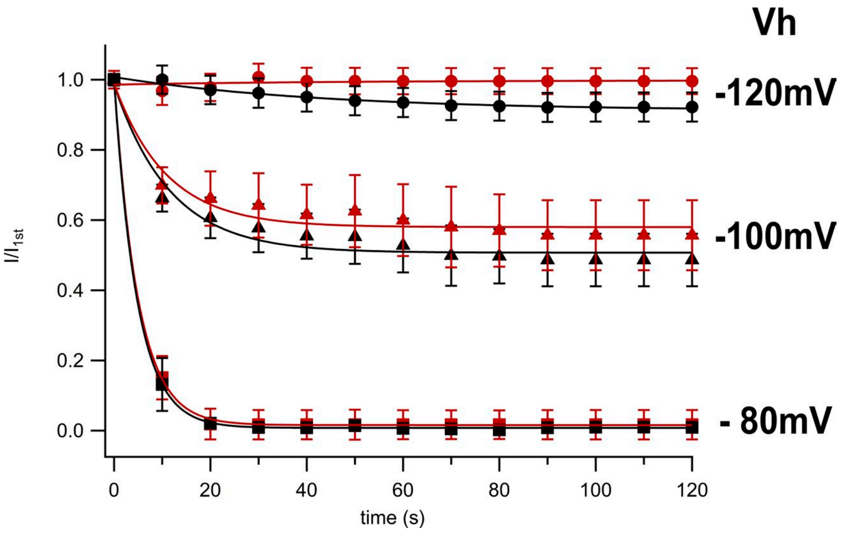

Figure S2. **Cumulative inactivation of NaChBac currents at different holding potentials.** Diary plot of NaChBac (NB) relative peak currents in control (black) and after BTX (red) modification, displayed for 300-ms pulses to −10 mV; 0.1 Hz; V_h = −120 mV (circles), −100 mV (triangles), and −80 mV (squares; *n* = 4 per condition).

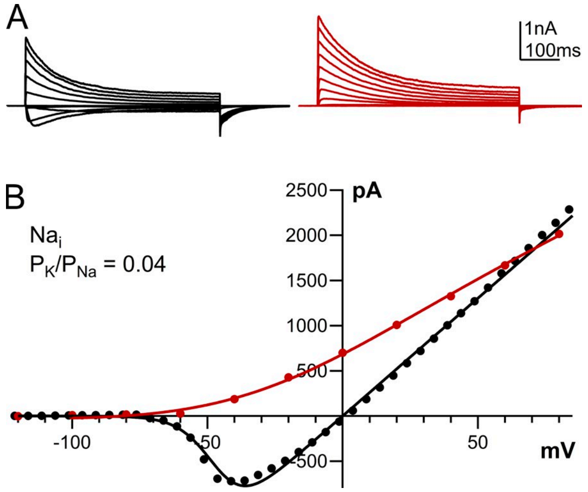

Figure S3. **Selectivity, calculated as relative permeability, P_K/P_{Na}, from the reversal potential. (A)** Whole-cell NaChBac currents in response to 500-ms activating pulses with 10 µM BTX in the pipette. Left: Symmetrical Na^+ (Na_i/Na_o, black). Right: Na_i/K_o (red). I-V is from −120 mV to 100 mV; V_h = −120 mV. **(B)** Peak *I–V* relationships for currents from A. Based on the shift of reversal potential, P_K/P_{Na} = 0.04 is approximately twofold higher than previously determined for unmodified NaChBac channels.

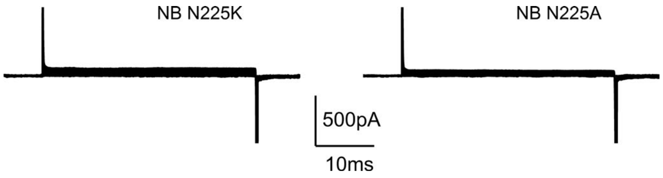

Figure S4. **NaChBac mutations (N225^{i20}K and N225^{i20}A) render the channel nonfunctional.** These mutations are homologous to the rNav1.4 domain 1 mutants N434^{i20}K and N434^{i20}A (see Fig. 5). Note that the homologous mutation in eukaryotic Nav channels replaces one amino acid change in a single domain and reduces the ability of BTX to modify the channel. In an assembled homotetrameric bacterial channel, this single mutation would result in four single-residue substitutions, one in each of the four subunits.

Table S1. **Partitioned energy of BTX interaction with NaChBac residues and sodium ions**

Residue	Universal label[a]	NaChBac position[b]	Energy, kcal/mol[c]
Thr	3i15	220	−2.32
Phe	3i16	221	−1.86
Phe	3i19	224	−1.77
Leu	2p49	190	−1.73
Thr	3p48	189	−1.72
Thr	2p48	189	−1.46
Phe	2i19	224	−1.05
Thr	2i15	220	−1.03
Leu	1p49	190	−1.02
Thr	1p48	189	−0.84
Val	3p47	188	−0.82
Val	1p47	188	−0.71
Thr	4i15	220	−0.67
Thr	4p48	189	−0.65
Ile	1i18	223	−0.63
Na^+		Na_{III}-Na_{IV}	−0.61
Thr	1i15	220	−0.50
Ile	3i18	223	−0.37
Leu	4p49	190	−0.33
Leu	3p49	190	+0.31
Ile	2i18	223	−0.30
Na^+		Na_I	+0.28
Phe	1i19	224	−0.25
Gln	3p45	186	−0.21
Val	4p46	187	−0.21
Phe	3i22	227	−0.21

[a]Prefixes 1, 2, 3, and 4 designate NaChBac subunits that correspond to repeat I, II, II, and IV, which in an extracellular view of the eukaryotic sodium channel are arranged clockwise (Dudley et al., 2000).
[b]Na_I and Na_{III} designate positions of sodium ions in the NavMs x-ray structure (Sula et al., 2017). Na_{IV} designates putative position of sodium ion at the focus of P1 helices (Tikhonov and Zhorov, 2017).
[c]Contributions with the absolute energy value <0.2 kcal/mol are not shown

Table S2. **Optimized parameters for the model shown in Fig. 8**

	Control		BTX			
	25 and 500 ms		25 ms		500 ms	
	α_o or β_o (/s)	*q(e)*	α_o or β_o (/s)	*q(e)*	α_o or β_o (/s)	*q(e)*
α1	13,000	3.1	7,700	2.6	1,100	2.7
β1	0.08		0.001		0.001	
α2	380	1.1	370	0	360	0
β2	6		200		110	
α3	24	0	13	0	8.5	0
β3	0.32		0.64	-1	9.5	-1
α4	0	0	9.2	0	27	0
β4	0		13		8.1	

In general, the time-dependent forward (α) and backward (β) transition rates are given by $\alpha(t) = \alpha_o \exp[q\ V(t)/(2\ k_b\ T)]$ and $\beta(t) = \beta_o \exp[-q\ V(t)/(2\ k_b\ T)]$, where $V(t)$ is the applied voltage at time t, T is temperature, k_b is Boltzmann's constant, α_o and β_o are the rates at zero applied voltage, and q is the charge moved within the membrane electric field up to the transition state intermediate. Note that we have made the simplifying assumption that the magnitude of q is the same for both forward and backward rates, which in general is not an absolute requirement. See also the kinetic modeling section in Materials and methods. In the simulations shown in Fig. 8, the following restrictions were applied. For control traces, the α3/β3 charges were held at zero, and the rate constants α4/β4, and their associated charges were set to zero. For BTX traces, all charges shown with a zero value were held at zero, while all other charges were allowed to vary.

References

Dudley, S.C. Jr., N. Chang, J. Hall, G. Lipkind, H.A. Fozzard, and R.J. French. 2000. mu-conotoxin GIIIA interactions with the voltage-gated Na(+) channel predict a clockwise arrangement of the domains. *J. Gen. Physiol.* 116:679–690. https://doi.org/10.1085/jgp.116.5.679

Sula, A., J. Booker, L.C. Ng, C.E. Naylor, P.G. DeCaen, and B.A. Wallace. 2017. The complete structure of an activated open sodium channel. *Nat. Commun.* 8:14205. https://doi.org/10.1038/ncomms14205

Tikhonov, D.B., and B.S. Zhorov. 2017. Mechanism of sodium channel block by local anesthetics, antiarrhythmics, and anticonvulsants. *J. Gen. Physiol.* 149:465–481. https://doi.org/10.1085/jgp.201611668
